# Quantitative proteomics reveals the novel co-expression signatures in early brain development for prognosis of glioblastoma multiforme

**DOI:** 10.18632/oncotarget.7416

**Published:** 2016-02-15

**Authors:** Xuexin Yu, Lin Feng, Dianming Liu, Lianfeng Zhang, Bo Wu, Wei Jiang, Zujing Han, Shujun Cheng

**Affiliations:** ^1^ College of Bioinformatics Science and Technology, Harbin Medical University, Harbin 150081, China; ^2^ State Key Laboratory of Molecular Oncology, Department of Etiology and Carcinogenesis, Cancer Institute and Hospital, Peking Union Medical College and Chinese Academy of Medical Sciences, Beijing 100021, China; ^3^ BGI Tech Solutions Co. Ltd., Beishan Industrial Zone, Yantian District, Shenzhen 518083, China; ^4^ Institute of Laboratory Animal Science, Chinese Academy of Medical Sciences and Peking Union Medical College, Beijing 100021, China; ^5^ Department of Histology and Embryology, School of Basic Medical Sciences, Capital Medical University, Beijing 100069, China

**Keywords:** brain development, co-expression, glioblastoma multiforme, prognosis, chemoresponse

## Abstract

Although several researches have explored the similarity across development and tumorigenesis in cellular behavior and underlying molecular mechanisms, not many have investigated the developmental characteristics at proteomic level and further extended to cancer clinical outcome. In this study, we used iTRAQ to quantify the protein expression changes during macaque rhesus brain development from fetuses at gestation 70 days to after born 5 years. Then, we performed weighted gene co-expression network analysis (WGCNA) on protein expression data of brain development to identify co-expressed modules that highly expressed on distinct development stages, including early stage, middle stage and late stage. Moreover, we used the univariate cox regression model to evaluate the prognostic potentials of these genes in two independent glioblastoma multiforme (GBM) datasets. The results showed that the modules highly expressed on early stage contained more reproducible prognostic genes, including ILF2, CCT7, CCT4, RPL10A, MSN, PRPS1, TFRC and APEX1. These genes were not only associated with clinical outcome, but also tended to influence chemoresponse. These signatures identified from embryonic brain development might contribute to precise prediction of GBM prognosis and identification of novel drug targets in GBM therapies. Thus, the development could become a viable reference model for researching cancers, including identifying novel prognostic markers and promoting new therapies.

## INTRODUCTION

Malignant gliomas are the most lethal and common brain tumor in adults. The most biologically aggressive subtype is glioblastoma multiforme (GBM) [1–4]. Its prognosis remains extremely poor [5, 6], even after the standard treatment for GBMs-surgical resection followed by adjuvant radiation therapy and chemotherapy [7], the median survival of these patients is only 15 months [8]. In past decades, substantial research effort has focused on identification markers of genetic alterations in GBMs that may associate with prognosis and may help to define subclasses of GBM patients [9, 10], such as TP53 mutation, EGFR mutation and PTEN mutation [11–13]. While these resources all have their own merit for uncovering the mechanism of malignant gliomas and promoting related therapies, they all focus on tumor samples and not concern other processes correlated with tumor progression such as brain development.

There are clear analogies between tumor and embryonic cells. The behavior of tumor cells in terms of growth, infiltration and suppression of immune system is similar to embryonic cells [14, 15]. Moreover, the mechanisms of antigenic loss, production of immunosuppressive cytokines and induction of apoptosis in infiltrating lymphocytes are the same as those found in embryonic cells [16, 17]. Thus, analysis the characteristics of brain developmental process may be able to provide useful information for the development of new integrated cancer therapeutic strategies and discovering novel prognosis markers. For example, the BrainSpan project has focused on studying transcriptional mechanisms involved in human brain development and providing the early roots of neurodevelopmental and psychiatric disorders [18]. At the present stage, they just obtain the transcriptome data, in consideration of the disagreement between mRNA level and protein level [19, 20], the proteome data could provide some novel and essential information.

Furthermore, for the developmental time series data, more and more researchers have found that co-expression module analysis could assist us to discovery a set of co-expression genes, which share a common function [21, 22]. Various studies have demonstrated the significance of co-expression genes in addressing biological problem [23, 24]. For instance, Jeremy A. and colleagues use weighted gene co-expression network analysis (WGCNA) to identify a set of modules for elucidating the details of human brain developmental mechanism [25].

We therefore hypothesized that the protein expression signatures co-expressed across brain development could provide more novel and essential information. In this study, we used isobaric tags for relative and solute quantitation (iTRAQ) technique [26] to quantify the relative protein expression level during macaque rhesus brain development from fetuses at gestation 45 days to after born 5 years. We firstly demonstrated that the protein expression profiles were better reflected the proteins' interaction compared with mRNA expression profiles from the co-expression perspective. Then, using the WGCNA package [27] in R, we found co-expression protein modules across brain developmental time points. These modules were highly expressed in different development stages and dominated distinct biological processes. Moreover, from the modules that were highly expressed in early brain development, we identified some genes that were associated with GBM patients overall survival in mRNA expression level and also involved in chemoresponse. Thus, these signatures derived from brain development may complement conventional clinical markers for outcome prediction, and may become new therapeutic targets in GBM therapy.

## RESULTS

### Summary of RNA-seq and iTRAQ Data

For nine rhesus macaque's samples, the proteome data were generated by iTRAQ of the above nine samples. Through searching Mascot (version 2.3.02), the final 1078 proteins were identified in the nine samples (protein expression data was deposited in Table S1).

The transcriptome data were obtained by RNA-seq by Illumina HiSeq^™^ 2000 sequencing platform. The clean reads were mapped to the rheMac3 genome by SOAP2 [28]. The gene expression level was normalized by converting the number of mapped reads per gene into RPKM that was stored in the Table S2.

**Figure 1:**
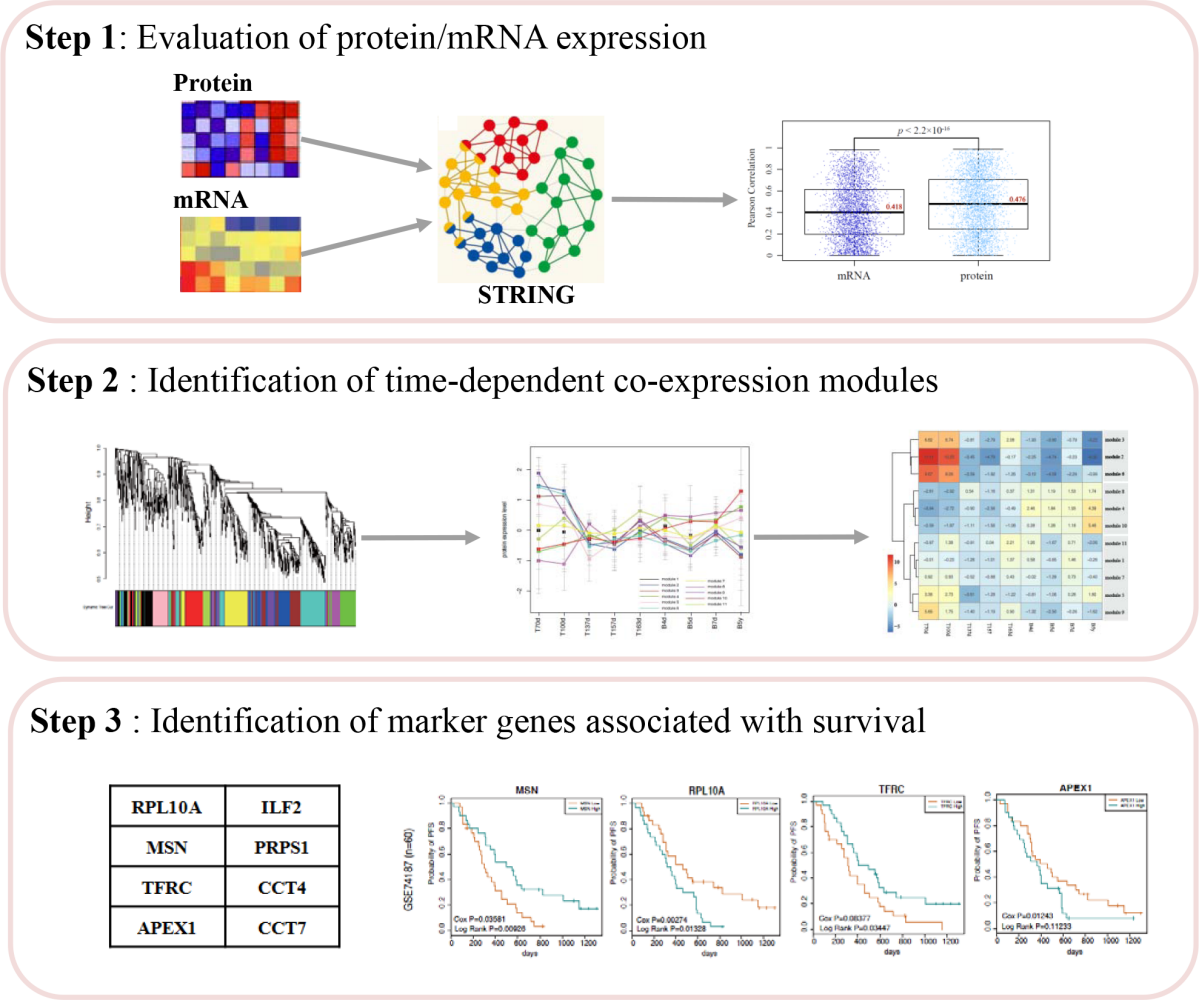
The workflow chart

Moreover, we performed the multi-step analysis to identify survival-associated signatures (Figure 1). The detailed information of each step was described in the following sections.

### Relationship between the protein/mRNA expression profiles and protein-protein interactions

**Figure 2:**
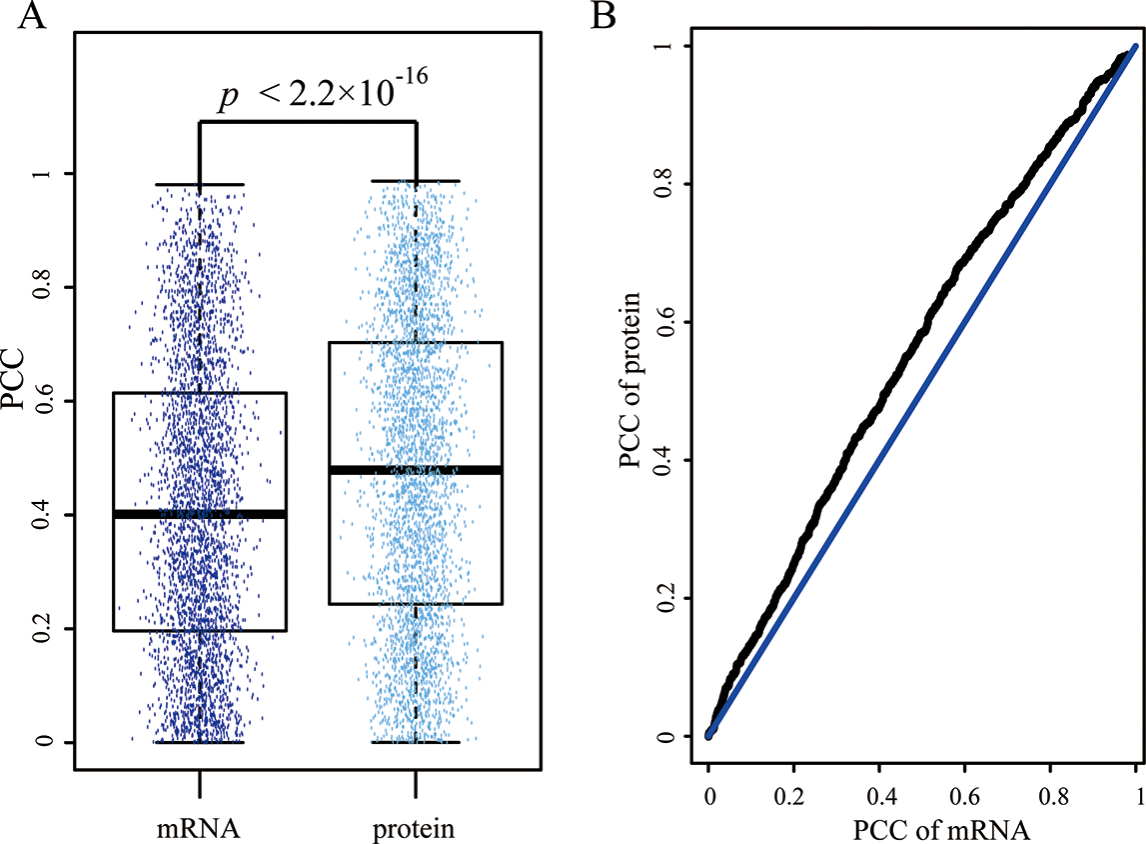
Disagreement of Pearson coefficient correlation (PCC) for each interaction in mRNA and protein expression level (**A**) The box plot represents the distribution of Pearson coefficient correlation for interactions in mRNA and protein expression level respectively. Each dot indicates the PCC of each interaction. (**B**) the Q-Q plot of PCCs in mRNA and protein expression level.

Through searching Mascot, the final 1078 proteins were identified in the nine samples used in the following analysis. Interacting proteins are more likely to be involved in similar biological functions and thus they are more likely to be co-expressed [29]. Therefore, we extracted 3544 interactions among 1078 proteins from STRING database (in Methods) and calculated the PCC for every interacted protein using the protein expression profiles, which contained 1078 proteins with 9 time points. For the same interacted proteins, we also computed their PCCs using the mRNA expression profiles. The mean PCC (*r_mean_* = 0.478) for protein expression profiles was higher than the mean PCC (*r_mean_* = 0.416) for mRNA expression profiles. Furthermore, the statistical significance for the difference between the above PCCs was measured by paired student *t*-test and the *P* value was less than 2.2 × 10^−16^ (Figure 2A).

We also used the quantitle-quantitle (Q-Q) plot to show the difference between the PCCs of mRNA and protein expression level (Figure 2B). The result suggested that the protein expression profiles were better reflected the proteins' interaction from the co-expression perspective.

### Identification of time-dependentco-expression modules

**Figure 3:**
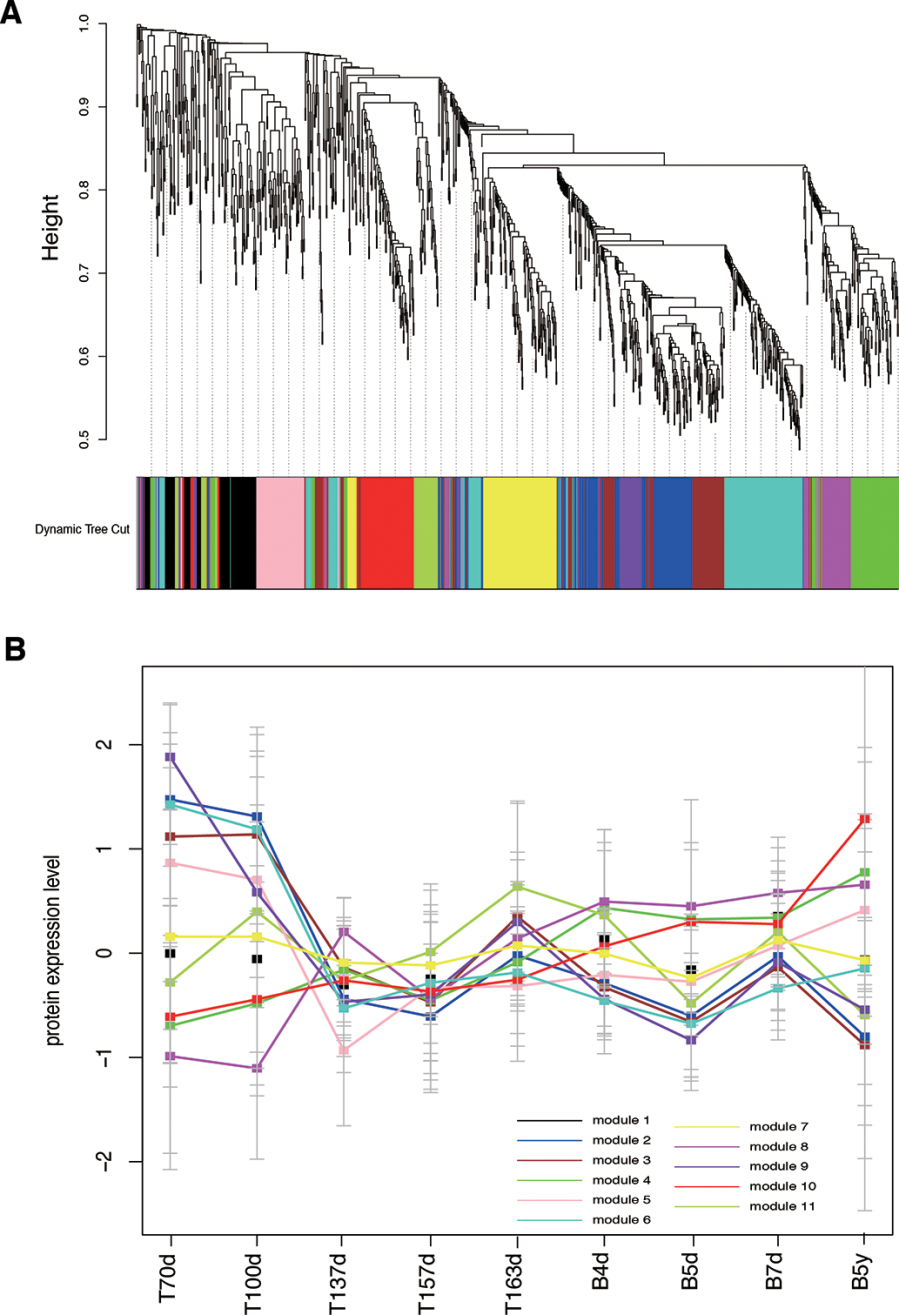
Co-expression analyses of brain development (**A**) WGCNA cluster dendrogram on 9 brain development samples groups proteins into 12 distinct modules (represents by different colors). (**B**) the 12 co-expression modules expression changes throughout the nine developmental time points, each module represents by one color that is the same as Figure 2.

To identify the principle features of the developing brain proteome, we performed weighted gene co-expression network analysis (WGCNA) on all 1078 proteins with nine time points, and identified 12 modules of co-expressed proteins (Figure 3A). WGCNA clustered proteins with similar expression patterns in an unbiased manner, allowing a biological interpretation of these patterns (biological process, disease and so on) [25, 30–32]. Here, to distinguish one module to another, each was assigned a number from 1 to 12. The modules ranged in size from 5 proteins in module 12 to 175 proteins in module 6. Moreover, we further filtered the proteins of each module and just reserved these proteins, which co-expressed in protein expression level and interacted with each other based on STRING database. The filtered modules could possess more significant biological sense. The original module 12 had 5 proteins, but these proteins did not interact with each other. Thus, the module 12 was omitted in the following analysis. The sizes of the rest 11 modules were shown in Table S3.

**Figure 4:**
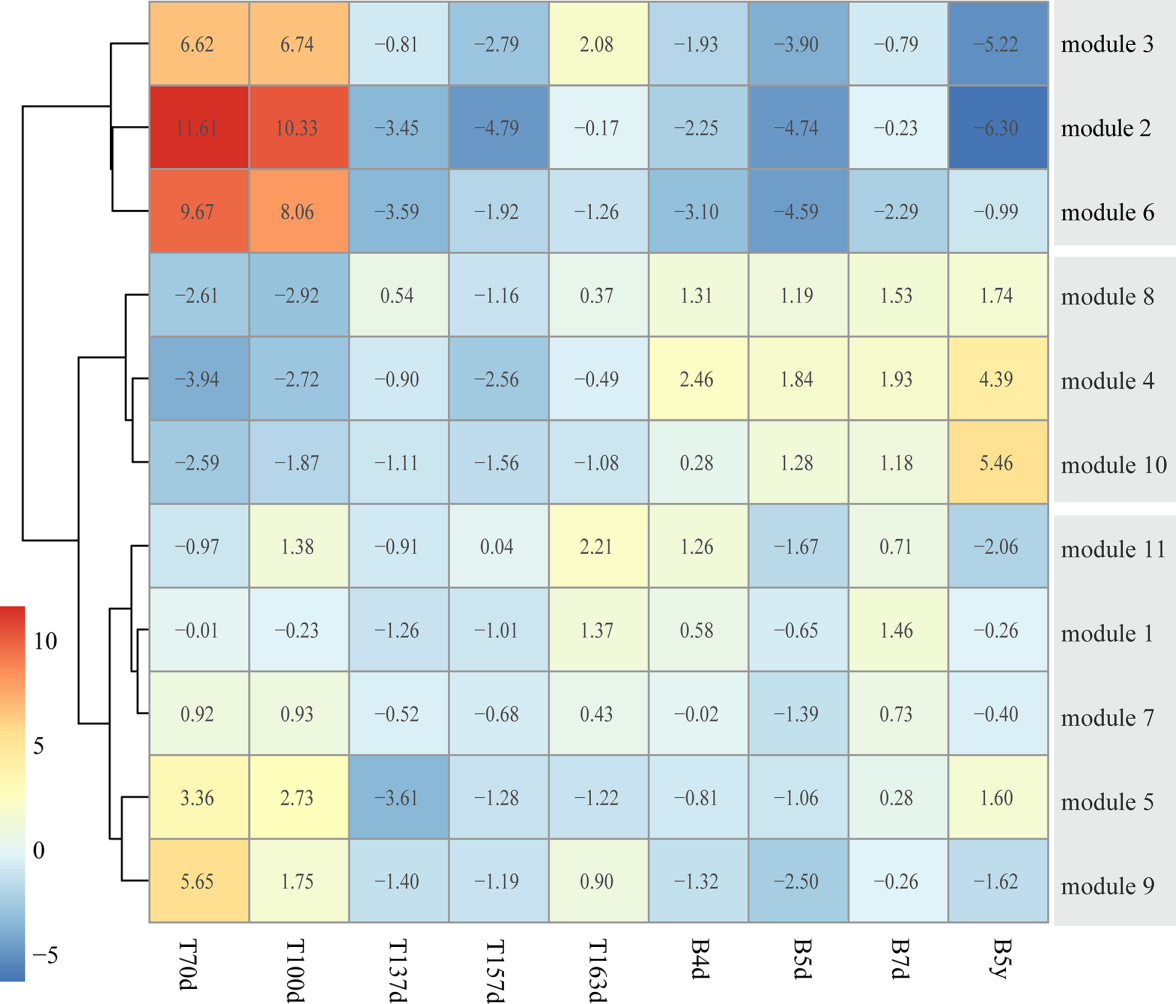
Clustering co-expression modules into different developmental stages The heatmap represents the three module groups and the color of each cell describes the mean expression value divided by the number of genes in this module.

The 11 modules had different expression patterns across the brain developmental time points (Figure 3B). In order to quantify the expression patterns, each module was scored to assess its activity in each time point, defined by averaging its protein expression values. Furthermore, we performed the hierarchical clustering on the activity matrix and we identified three groups of modules, including the first group was highly expressed at early brain development (module 2, 3 and 6, named early group), the second group was highly expressed after birth (module 4, 8 and 10, named late group), and the third group was a mixed group as transition (module 1, 5, 7, 9 and 11, named middle group) (Figure 4). Here, we used DAVID [33, 34] to find the biological process (BP) terms of genes in each module. As a result, we found that the genes of modules in three groups dominated different biological processes (Table S4). For example, module 6 contained proteins associated with neuron recognition, neural tube closure, primary neural tube formation, and positive regulation of neuron differentiation. The proteins of module 4, 8, and 10 tended to highly expressed after birth, and the functions of these three modules were associated with some brain disorders, such as Huntington's disease, Parkinson's disease, and Alzheimer's disease. Moreover, the functions of the proteins in module 1 contained synaptic transmission, regulation of neurological system process, and regulation of neurological system process.

### Co-expression modules of early brain development associated with survival in patients with GBM

**Figure 5:**
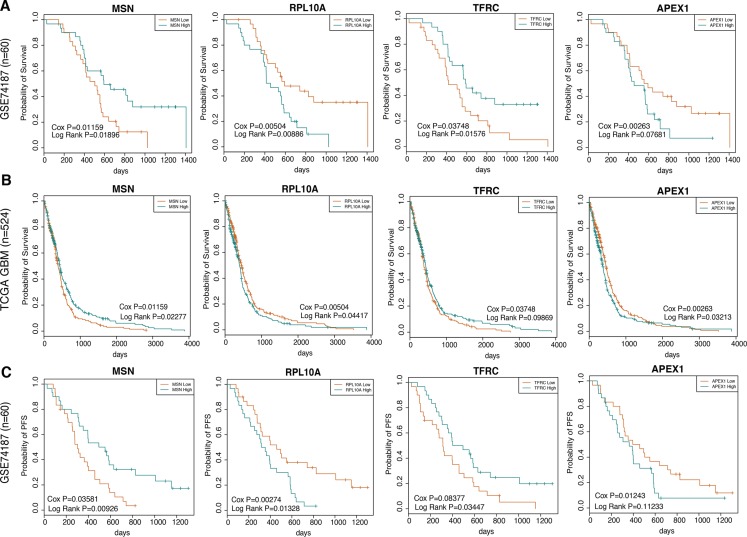
The co-expression signatures in early brain development predictsOS as well as PFS in GBM patients (**A**) and (**B**) shows representative OS curves based on data analyzed from GSE74187 and TCGA GBM datasets respectively. (**C**) shows respective representative PFS curves based on data analyzed from GSE74187.

Based on the above three groups module genes, we further tested whether these genes had predictive power in clinical outcome among GBM patients in two independent data sets including GSE74187 and TCGA GBM data, wherein the 60 GBM samples in GSE74187 were collected by ourselves. For GSE74187 dataset, we performed univariate cox regression model to evaluate the significance of the correlations between individual gene expression and overall survival (OS) and identified 18, 11 and 17 genes significantly (*P* < 0.05) related with overall survival time, in early, middle and late group respectively. In order to verify the reproducibility, we then validated the prognostic impact of these significantly genes in one independent GBM set by the same method and parameter, namely the TCGA GBM set (*n* = 524). As a result, there were 8, 4 and 3 genes showed consistent correlations between their expression and overall survival. Notably, the early group statistically significant contained the survival-associated genes (*P* = 0.0086 in 10000 permutations), and the eight genes were ILF2, CCT7, CCT4, RPL10A, MSN, PRPS1, TFRC and APEX1 (Table S5). For each gene, we divided the samples into two groups, including high group and low group, based on the median gene expression value. Furthermore, we performed log rank test to evaluate the difference of overall survival in the two group samples (Figure S1). Representative graphs from the eight genes were presented in Figure 5A and 5B. We observed that that the eight genes were capable of accurately stratifying patients according to expression level for each gene. We also used univariate cox regression model with the same parameter to evaluate the association between these eight genes expression and progression-free survival (PFS) in GSE74187, we found the six of eight genes were significant (*P* < 0.05), while the rest two genes' expression were also weakly related with PFS (0.05 < *P* < 0.1) in Figure 5C.

**Figure 6:**
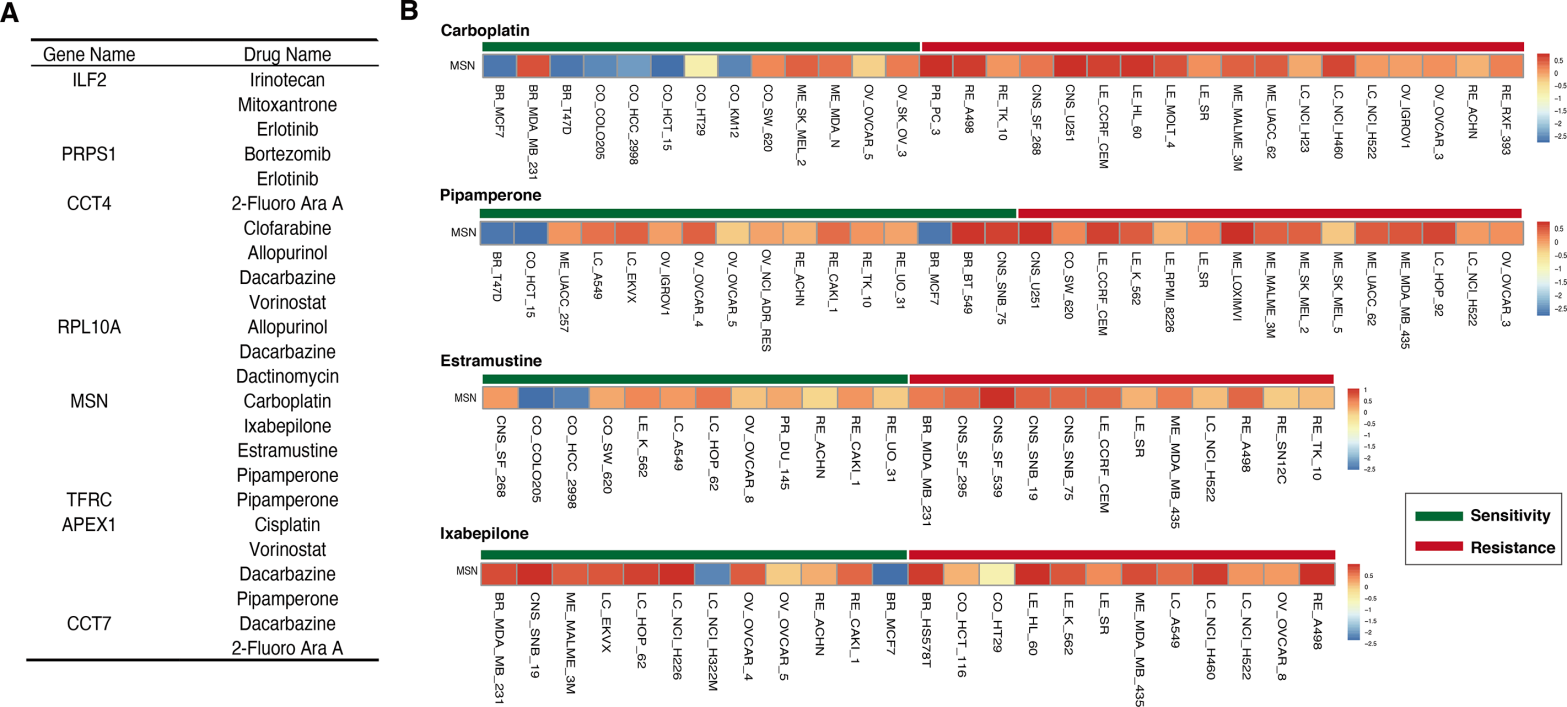
Signature genes associated with some drugs' chemoresponse (**A**) The eight genes associates with different number drugs' chemoresponse. (**B**) The example of MSN expression level in sensitive and resistant cell lines for four drugs, including carboplatin, pipamperone, estramustine and ixabeilone.

Here, TFRC and APEX1 were the known drug targets of ganite and lucanthone that were approved by FDA respectively. Particularly, lucanthone was used as a radiation sensitizer in the treatment of brain cancer [35, 36]. In order to evaluate the effect of the eight genes for the mode of action of drugs, we used NCI-60 data to assess whether these genes' expression was associated with chemoresponse (see in Materials and Methods). In Figure 6A, we showed the eight genes and the associated drugs. For example, the expression level of MSN was related with four drugs' chemoresponse, including Carboplatin, Pipamperone, Estramustine and Ixabepilone (Figure 6B). Importantly, Carboplatin was used to treat the central nervous system tumor [37, 38]. Furthermore, we also found that some genes were related with the same drug's chemoresponse. Thus, we deduced that the eight genes might be potential drug targets for brain tumors and these drugs might be repositioned for brain tumor treatment.

## DISCUSSION

Comparing with the transcriptome data, studying the developing macaque rhesus brain in protein expression level could provide more novel and essential information for elucidating the details of brain formation and function, and for understanding developmental mechanisms underlying brain disorders such as autism and brain tumor. The similarities between tumor and pregnancy go further than the mechanism of immune escape and predictably extend to intermediate metabolism process [14, 27]. Thus, a careful study of the well-known mechanisms of development used by the embryo could provide fresh ideas on designing new cancer therapies. Several recent studies have focused on this issue and explored the relationship between development and tumor on gene expression level [39]. At present, these researches have only investigated the developmental process on transcriptome level. While we use iTRAQ to measure the protein expression during the macaque rhesus brain development from fetuses at gestation 70 days to after born 5 years. This data resource could complement the present brain development data pool.

In this study, we utilized the protein expression data during macaque rhesus brain development to depict the brain function formation and changes by co-expression modules. We also found that the protein expression of early developmental stage was significantly positive correlated with the mRNA expression of GBM. Furthermore, we found that the protein expression better reflected the proteins' interaction compared with mRNA expression. Importantly, the modules that highly expressed on early brain development contained more markers associated with GBM patient's outcome. We not only performed univariable cox regression, but also used multivariate cox regression model for adjusting the age, gender and race information, the eight genes were significant in both two regression models. These findings might provide fresh ideas for understanding GBM and providing new therapy targets. In particular, the low expression level of MSN associated with poor prognosis in GBM patients, and also related with chemoresponse of Carboplatin, so we deduced that Carboplatin could use for treating the GBM patients with lower MSN expression level. Moreover, the method of the drug sensitivity analysis could be used in similar project to explore whether the genes have influence on the drug sensitivity.

With the increasing volume of developmental data, the analysis of tumor and development relationships in methylation or non-coding RNA level will be considered in the future works. It is our expectation that the study of development–cancer associations will provide fresh ideas for understanding tumor pathological systems and ultimately improve therapeutics.

## MATERIALS AND METHODS

### Rhesus macaques and samples

The present study was approved by the ethics committee at Institute of Laboratory Animal Science, Chinese Academy of Medical Sciences (CAMS) & Peking Union Medical College (PUMC). All experiments were performed in accordance with relevant guidelines and regulations. Rhesus macaques at nine different developmental time points, including fetuses at gestation 70 days, 100 days, 137 days, 157 days, 163 days (T70d, T100d, T137d, T157d, T163d), after born 4 days, 5 days, 7 days (B4d, B5d, B7d) and after born 5 years (B5y). All rhesus were raised at the Institute of Laboratory Animal Science, Chinese Academy of Medical Sciences (CAMS) & Peking Union Medical College (PUMC). Caesarean sections were performed to obtain fetuses from pregnant rhesus at given developmental time point. Organ was isolated by manual dissection after euthanasia of living animals. For younger fetuses, dissection was completed with the aid of a dissecting microscope (Nikon, Japan). The brain samples from each rhesus were individually prepared for RNA and protein extraction.

### Protein extraction and peptide labeled by iTRAQ

The harvested cells were lysed in the buffer, containing 8 M urea, 4% CHAPS, 10 mM dithiotreitol, and 40 m MTris-HCl, pH 8.0, with sonication in ice. After centrifugation at 12,000 g at 4°C, the supernatants were reduced and alkylated by 10 mM dithiotreitol and 55 mM iodoacetamide. The treated proteins were precipitated in 80% acetone at −20°C overnight, and the precipitants were re-suspended in 0.8 M urea and 500 mM tetraethylammonium bicarbonate (TEAB), pH 8.5. The protein concentrations were determined using the Bradford method followed by a 16 h trypsin digestion at 37°C. The tryptic peptides were labeled by the 8-plex iTRAQ reagents (AB Sciex, Foster City, CA) following the manufacturer's protocol. After 2 h of labeling reactions, the reaction solvents were removed by Speed-vacuum, and the labeled peptides were dissolved in 20 mM NH4FA, pH 10, for the following experiments.

### Database searches for peptide and protein identification

The 2.3.02 version of Mascot software (Matrix Science, Boston, MA) was used to simultaneously identify and quantify proteins. In this version, only unique peptides used for protein quantification were chosen to quantify proteins more precisely. Searches were made against the NCBI Macaca protein database (53990 sequences). Spectra from the 12 fractions were combined into one MGF (Mascot generic format) file after the raw data were loaded, and the MGF file was searched. The search parameters were: i) trypsin was chosen as the enzyme with one missed cleavage allowed; ii) the fixed modifications of carbamidomethylation were set as Cys, and variable modifications of oxidation as Met; iii) peptide tolerance was set as 0.05 Da, and MS/MS tolerance was set as 0.1 Da. The peptide charge was set as Mr, and monoisotopic mass was chosen. An automatic decoy database search strategy was employed to estimate the false discovery rate (FDR). The FDR was calculated as the false positive matches divided by the total matches. In the final search results, the FDR was less than 1.5%. The iTRAQ 8-plex was chosen for quantification during the search. The search results were passed through additional filters before data exportation. For protein identification, the filters were set as follows: significance threshold P, 0.05 (with 95% confidence) and ion score or expected cutoff less than 0.05 (with 95% confidence). For protein quantitation, the filters were set as follows: “median” was chosen for the protein ratio type (http://www.matrixscience.com/help/quant_config_help.html); the minimum precursor charge was set to 2 and minimum peptides were set to 2; only unique peptides were used to quantify proteins. The median intensities were set as normalization, and outliers were removed automatically. The peptide threshold was set as above for identity.

At last, there were 1078 proteins with protein expression at each sample.

### GBM patient gene expression and clinical data

Sixty GBM samples with OS and PFS information were obtained during surgical resection from Tian Tan Hospital from 2008 to 2010. All donors signed informed consent forms. The use of human tissue samples and the experimental procedures for this study were reviewed and approved by the Ethics Committee of the Cancer Institute and Hospital, Chinese Academy of Medical Sciences.

Total RNA was isolated with Trizol reagent (Invitrogen, CA, USA), and those allocated for microarray detection were purified with an RNeasy kit (Qiagen, MD, USA). RNA was quantitated with ND-1000 UV-VIS Spectrophotometer (NanoDrop Technologies, DE, USA) and the integrity of RNA was assessed using the RNA 6000 Labchip kit in combination with the Agilent 2100 Bioanalyzer (Agilent, CA, USA). Purified total RNA samples were labeled and hybridized to Agilent 4 × 44 K Whole Human Genome Oligo Microarrays according to the manufacturer's instructions.

Furthermore, the raw and processed gene expression data and clinical information of the sixty GBM samples have been deposited in Gene Expression Omnibus (GEO) database with the series accession numbers GSE74187.

### Protein-protein interaction data

The rhesus macaque protein-protein interaction (PPI) data were obtained from STRING database v9.1. We obtained 3544 interactions among the 1078 proteins that are experimentally verified (experimental scores > 200) and have total scores (> 400).

### Quantification of the relationship between protein/mRNA expression and PPI

We used Pearson correlation coefficient (PCC) as the measure of relationship between protein/mRNA expression and protein interactions. For the 3544 interactions, we computed the PCC for each interaction in protein and mRNA expression profiles respectively.

In order to quantitative comparison the PCC in protein expression profiles with the PCC in mRNA expression profiles, we used paired student *t*-test to determined whether the above two PCC sets were different. If the *P* value was less than 0.05, it suggests that the two data sets were significantly different.

### Identification of co-expression modules by WGCNA

We used the WGCNA package in R to construct unsigned co-expression network. There were 1078 proteins in this network. Network construction was performed using blockwiseModules function in the WGCNA. For each set of genes, pair-wise correlation matrix was computed, and adjacency matrix was calculated by raising the correlation matrix to a power (power = 10). The topological overlap measure was used to measure the network interconnectedness, which was calculated based on the adjacency matrix. Furthermore, the topological overlap based dissimilarity was then used as input for average linkage hierarchical clustering. Finally, modules were identified on the dendrogram using Dynamic Tree Cut algorithm [40].

### Survival analysis

Univariable Cox Proportional Hazards regression model was used to evaluate the association of a given gene expression with survival. *P* values < 0.05 were considered statistically significant. The above analysis was performed using the R package “survival”.

### Determination of the gene expression associated with chemoresponse

For NCI-60, the 60 cell lines were previously assayed for their responses to a variety of compounds, which were measured by the IC_50_. For each compound, the log10 (IC_50_) values were normalized across the 60 cell lines. Cell lines with value of log10 (IC_50_) that were greater than μ + SD were defined as resistant to the compound, whereas those with log10 (IC_50_) values that were less than μ−SD were regarded as sensitive. Cell lines with values for log10 (IC_50_) within μ ± SD were considered to be intermediate and were then eliminated from further analysis. The following analysis was performed for compounds that had at least 10 sensitive and 10 resistant cell lines. Of the 20,503 compounds evaluated in Developmental Therapeutics Program (DTP), 5,688 met these criteria, including 38 drugs that were approved by FDA.

For the 38 FDA approved drugs, we used *t*-test to determine whether the gene differential expressed between sensitive and resistant cell lines for each drug. If the *P* value < 0.05, we defined this gene expression was associated with chemoresponse of the given drug.

## SUPPLEMENTARY MATERIALS FIGURE AND TABLES








