# Supplementary material for: Quantitative proteomics reveals the novel co-expression signatures in early brain development for prognosis of glioblastoma multiforme
Source: Oncotarget. 2016 Feb 15;7(12):14161–71. doi: 10.18632/oncotarget.7416 (PMC4924705; doi:10.18632/oncotarget.7416)
Supplement: Supplementary file 1 [file oncotarget-07-14161-s001.pdf]

Quantitative proteomics reveals the novel co-expression signatures in early brain development for prognosis of glioblastoma multiforme

Supplementary Materials

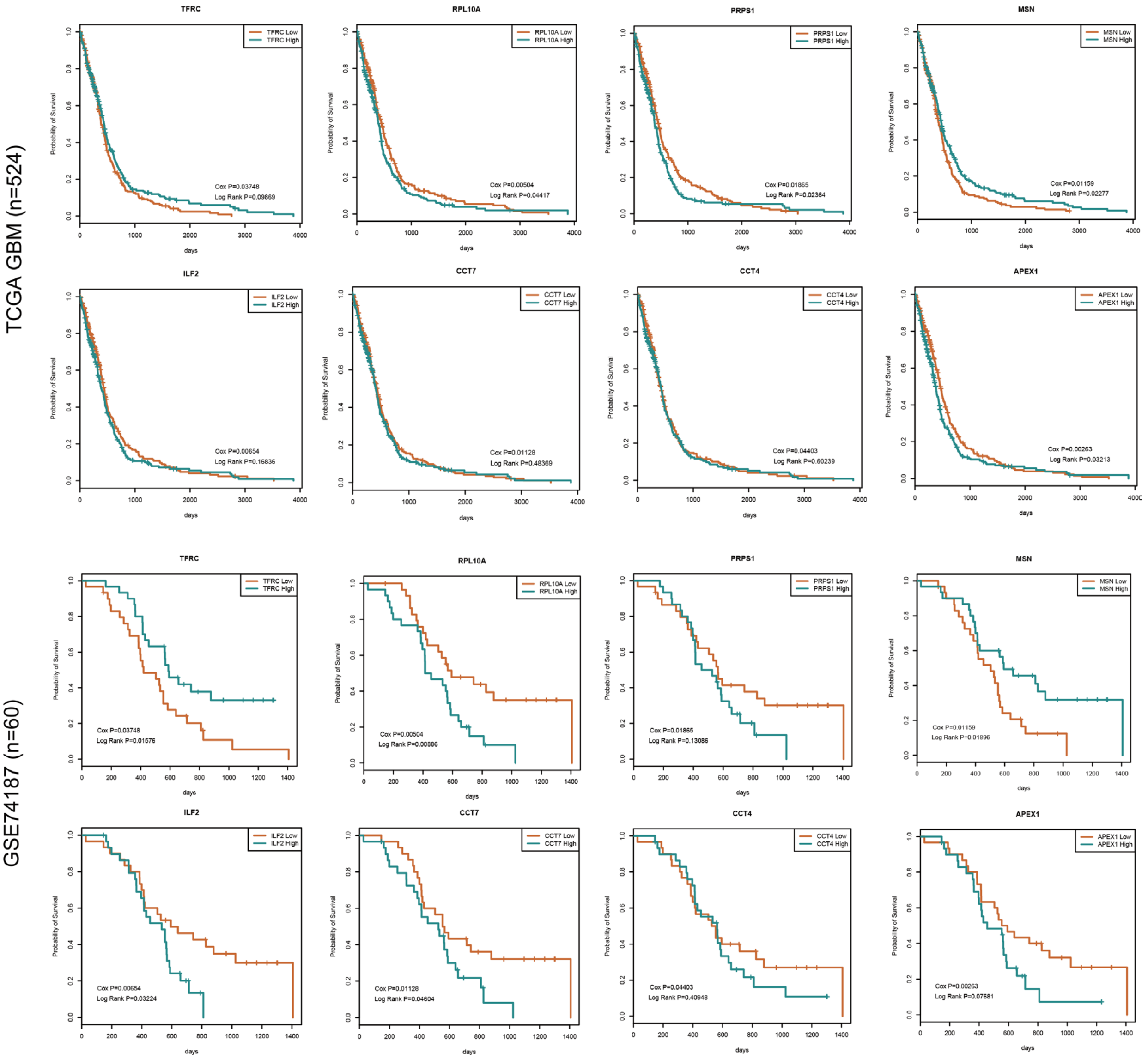

Supplementary Figure S1: The survival curves of eight marker genes.

Supplementary Table S1: The protein expression profile

Supplementary Table S2: The mRNA expression profile

Supplementary Table S3: The number of genes in each module

	**module 1**	**module 2**	**module 3**	**module 4**	**module 5**	**module 6**	**module 7**	**module 8**	**module 9**	**module 10**	**module 11**	**module 12**
NO.edge of original co-expression network	4005	8256	7260	5356	2628	15225	6786	2346	1431	4095	1225	10
NO.node of original co-expression network	90	129	121	104	73	175	117	69	54	91	50	5
NO.edge of filtered network	16	94	34	30	13	44	32	4	6	12	12	0
NO.node of filtered network	17	62	35	32	15	46	34	7	9	18	12	0

#The table described the original module size, including the number of node and edge. Moreover, the table also represented the filter module size which was filtered by PPI from STRING database.

Supplementary Table S4: The GO and pathway enrichment for each module

Supplementary Table S5: The function of eight marker genes

Gene	Function	PMID/DOI
ILF2	IL2 and NF90 proteins formed an NFAT DNA-binding activity that was enhanced by T-cell stimulation and inhibited by cyclosporin A and FK56. Moreover, combination of temozolomide with immunocytokine F16–IL2 for the treatment of glioblastoma.	7519613<br>20736949
PRPS1	PRPS1 encodes an enzyme that catalyzes the phosphoribosylation of ribose 5-phosphate to 5-phosphoribosyl-1-pyrophosphate, which is necessary for purine metabolism and nucleotide biosynthesis. Moreover, negative feedback-defective PRPS1 mutants drive thiopurine resistance in relapsed childhood ALL.	25962120
TFRC	TFRC is necessary for development of erythrocytes and the nervous system. Moreover, this gene expression is associated with shorter survival in GBM patients.	23502430
MSN	MSN appears to function as cross-linkers between plasma membranes and actin-based cytoskeletons. Moreover, MSN in hyaluronan induces cell migration in glioblastoma multiforme.	23855374
RPL10A	RPL10A belongs to the L1P family of ribosomal proteins. It is located in the cytoplasm. Studies in mice have shown that the expression of the ribosomal protein L10a gene expressed in neural precursor cells during development.	16646669
CCT7	CCT7 is essential for CCNE1 maturation and accumulation.	9819444
APEX1	The two major activities of APEX1 in DNA repair and redox regulation of transcriptional factors. Moreover, increased expression of APEX1 gene coding for Ape1 positively correlates with better survival of glioblastoma patients.	doi:10.1158/1557-3265.<br>PMS14-B26
CCT4	CCT4 enhances the binding of TARBP1 and RNA polymerase II to TAR. Diseases associated with CCT4 include sensory peripheral neuropathy and hereditary sensory neuropathy.	7298497<br>12874111
